# Drug-Induced Rhabdomyolysis with Elevated Cardiac Troponin T

**DOI:** 10.1155/2015/270204

**Published:** 2015-10-08

**Authors:** Gro Egholm, Manan Pareek

**Affiliations:** ^1^Department of Cardiology, Aarhus University Hospital, Skejby, Palle Juul-Jensens Boulevard 99, 8200 Aarhus N, Denmark; ^2^Cardiovascular and Metabolic Preventive Clinic, Department of Endocrinology, Odense University Hospital, Sdr. Boulevard 29, 5000 Odense C, Denmark

## Abstract

The essential role of cardiac troponin in the diagnosis of acute myocardial infarction has led to the development of high-sensitivity assays, which are able to detect very small amounts of myocardial necrosis. The high-sensitivity cardiac troponin T assay, however, is not entirely specific for myocardial injury. This case report describes a 48-year-old woman, who, two years after cardiac transplantation, presented with rhabdomyolysis. During the course of the disease, her troponin T level was elevated on repeated occasions, but other definitive evidence of myocardial injury was not found. Asymptomatic cardiac troponin T elevations during rhabdomyolysis may be due to either cardiac involvement or false positive results stemming from skeletal muscle injury.

## 1. Introduction

Myocardial injury is defined as the disruption of normal cardiac myocyte membrane integrity leading to release of intracellular components. These include structural proteins such as troponin (Tn) and creatine kinase (CK). The essential role of these myocardial tissue-specific cardiac biomarkers in the diagnosis of acute myocardial infarction (MI) has led to the development of high-sensitivity cardiac TnT (hs-cTnT) and I (hs-cTnI) analyses. The improved sensitivity, however, comes at the cost of a reduced specificity for MI, as these new assays have the ability to detect myocardial necrosis associated with a large number of other conditions. Furthermore, the hs-cTnT assay is not entirely specific for myocardial injury, and as with any other test, false positive and negative results do occur [1–3]. Therefore, correct interpretation of elevated cardiac biomarkers can be challenging. We present a case in which the elevated cTnT levels probably were not due to myocardial damage.

## 2. Case Presentation

A 48-year-old woman, who had undergone cardiac transplantation two years earlier, was admitted to the department of cardiology due to diarrhea, fatigue, and malaise. The patient was treated with the usual immunosuppressive regimen, comprising tacrolimus 2 mg b.i.d., mycophenolate mofetil 500 mg b.i.d., and prednisone 5 mg q.d. She further received atorvastatin due to concomitant dyslipidemia. A few months prior to admission, the dose of atorvastatin had been increased from 40 mg to 80 mg daily.

Blood tests at admission showed elevated levels of creatinine at 612 *μ*mol/L (reference range 45–90 *μ*mol/L), CK 18,934 U/L (50–150 U/L), and myoglobin 11,718 *μ*g/L (<75 *μ*g/L). The symptoms and findings were consistent with a diagnosis of rhabdomyolysis with acute kidney injury, and a regimen of forced alkaline diuresis was initiated. The condition was ascribed to the dose increase of atorvastatin and the possible interaction between this drug and tacrolimus, resulting in a reduced elimination of atorvastatin [4]. Consequently, atorvastatin was discontinued, and the dose of tacrolimus was reduced.

On the seventh day of admission, elevated levels of hs-cTnT at 471 ng/L (99th percentile 14 ng/L) and CK isoenzyme MB (CK-MB) 162 *μ*g/L (<3 *μ*g/L) alongside increasing levels of CK 30,750 U/L and myoglobin 29,120 *μ*g/L were found. Subsequent serial measurements showed increasing levels of cTnT, but decreasing levels of CK-MB, CK, myoglobin, and creatinine. Furthermore, the CK-MB/CK ratio increased from less than 1% initially to 12%. Due to the elevated cardiac biomarkers, serial electrocardiograms, echocardiograms, and an endomyocardial biopsy were performed, which were all unremarkable. A peripheral muscle biopsy showed toxic myopathy, but no signs of inflammatory myositis. The patient was discharged after a total of four weeks. The statin treatment was not resumed, and she has not shown any signs of relapse since.

## 3. Discussion

Pathologically, an MI is defined as myocardial cell death due to prolonged ischemia. Therefore, the diagnosis of an acute MI requires evidence of myocardial necrosis in a clinical setting consistent with myocardial ischemia. Myocardial necrosis is defined as a rise and/or fall of cardiac biomarker values (preferably cTn) with at least one value above the 99th percentile upper reference limit, whereas myocardial ischemia is defined as at least one of the following: (1) symptoms of ischemia, (2) new or presumed new significant ST-segment or T-wave changes or new left bundle branch block, (3) development of pathological Q waves in the ECG, (4) imaging evidence of new loss of viable myocardium or new regional wall motion abnormality, or (5) identification of an intracoronary thrombus by angiography or autopsy [5].

The Tn complex is part of the contractile system of skeletal and cardiac muscle and consists of three subunits: TnC, TnI, and TnT. Cardiac TnC cannot be used as a cardiac biomarker due to its expression in adult skeletal muscle, whereas cTnI and cTnT are almost exclusively expressed in cardiomyocytes. However, cTnT is regularly found in embryonic skeletal muscle, and its messenger RNA (mRNA) is sometimes reexpressed in patients with skeletal muscle disease. These TnT isoforms are similar to those of normal and diseased myocardium and seem to be detected by the antibodies used in cTnT assays due to cross-reaction [6, 7]. Although cTnI mRNA is also expressed in skeletal muscle myopathies, these isoforms apparently go undetected by the contemporary cTnI assays, enabling one to distinguish between the presence and absence of myocardial injury [7–9].

It has been suggested that cTnT elevations detected during rhabdomyolysis may be the result of reexpression of a specific isoform seen in regenerating skeletal muscle, as this condition is characterized by simultaneous degradation and regeneration of skeletal muscle [10]. The elevated cTnT levels in this case could therefore be due to regeneration of skeletal muscle tissue and not myocardial damage, which was also reflected in the increasing cTnT-levels despite decreasing levels of CK and myoglobin and particularly the increasing CK-MB/CK ratio [11]. Furthermore, cardiac involvement during rhabdomyolysis has been described previously, whereby cTn elevations should not necessarily be ignored, but at the same time, the risk of false positive cTnT results increases with severe skeletal muscle injury [12]. Unfortunately, cardiac magnetic resonance imaging (MRI) was not done, although this modality might have provided a more definite answer, for example, if the patient had more diffuse injury due to myocarditis or rhabdomyolysis [13].

In the few similar cases described in the literature, the definitive evidence of cardiac involvement during drug-induced rhabdomyolysis was provided by autopsy [12, 14, 15]. Furthermore, Punukollu et al. reviewed 91 patients with rhabdomyolysis of whom 19 had elevated cTnI; however, none of these patients had segmental wall motion abnormalities on echocardiography [16].

This case report highlights the importance of interpreting the results of the hs-TnT assay correctly and taking into account the diagnostic criteria when designating a diagnosis of MI: relevant symptoms, ECG-changes, or imaging findings as well as the compulsory rise and fall pattern in cTn. In addition, in patients with rhabdomyolysis, serial CK-MB/CK ratio, cTnI, and cardiac MRI may be helpful in order to differentiate between cardiac involvement and false positive cTnT results.
